# Correction: Bartha et al. How to Measure Adherence to a Mediterranean Diet in Dental Studies: Is a Short Adherence Screener Enough? A Comparative Analysis. *Nutrients* 2022, *14*, 1300

**DOI:** 10.3390/nu14091845

**Published:** 2022-04-28

**Authors:** Valentin Bartha, Lea Exner, Anna-Lisa Meyer, Maryam Basrai, Daniela Schweikert, Michael Adolph, Thomas Bruckner, Christian Meller, Johan Peter Woelber, Diana Wolff

**Affiliations:** 1 Department for Conservative Dentistry, University Hospital of Heidelberg, Im Neuenheimer Feld 400, 69120 Heidelberg, Germany; diana.wolff@med.uni-heidelberg.de; 2 Department for Conservative Dentistry, University Hospital Tuebingen, Osianderstraße 2-8, 72076 Tübingen, Germany; exner.lea@gmx.de (L.E.); christian.meller@med.uni-tuebingen.de (C.M.); 3 Institute of Nutritional Medicine, University of Hohenheim, Fruwirthstr. 12, 70599 Stuttgart, Germany; anna94.meyer@yahoo.de (A.-L.M.); m.basrai@uni-hohenheim.de (M.B.); 4 Department of Nutrition Management and Nutrition Support Team, University Hospital Tuebingen, Hoppe-Seyler-Straße, 72076 Tübingen, Germany; daniela.schweikert@med.uni-tuebingen.de (D.S.); michael.adolph@med.uni-tuebingen.de (M.A.); 5 Institute of Medical Biometry, Faculty of Medicine, University of Heidelberg, Im Neuenheimer Feld 130.3, 69120 Heidelberg, Germany; bruckner@imbi.uni-heidelberg.de; 6 Department of Operative Dentistry and Periodontology, Faculty of Medicine, University of Freiburg, Hugstetter Str. 55, 79106 Freiburg, Germany; johan.woelber@uniklinik-freiburg.de

## Error in Figures

The authors would like to make a correction in a recently published paper [1]. There were errors in Figures 1, 2 and 4. In the original Figures, there are missing lines, arrows, boxes, colours and confidence intervals due to the incompatibilities between different computer operating systems.

Original Figure 1:


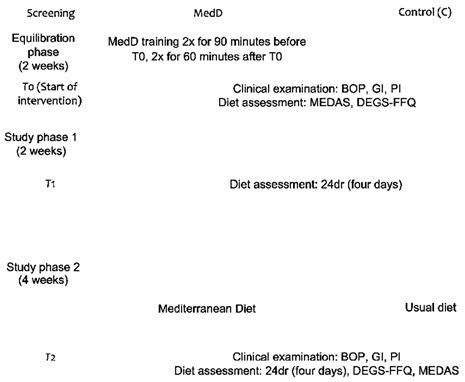


We would like it to be corrected as shown below.

New Figure 1:


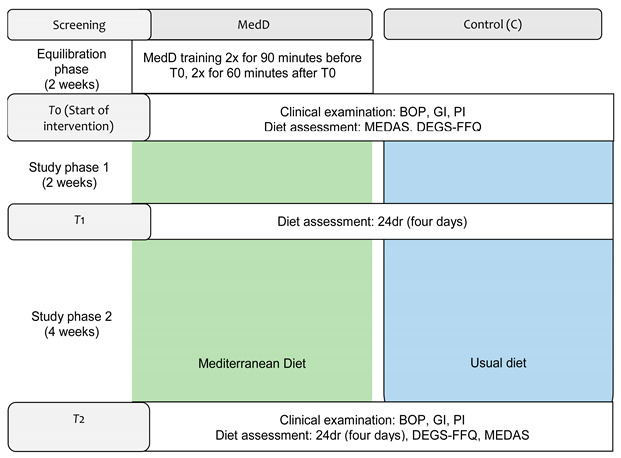


Original Figure 2:


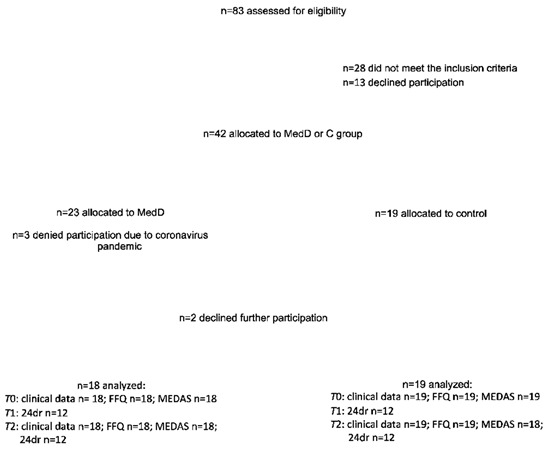


New Figure 2:


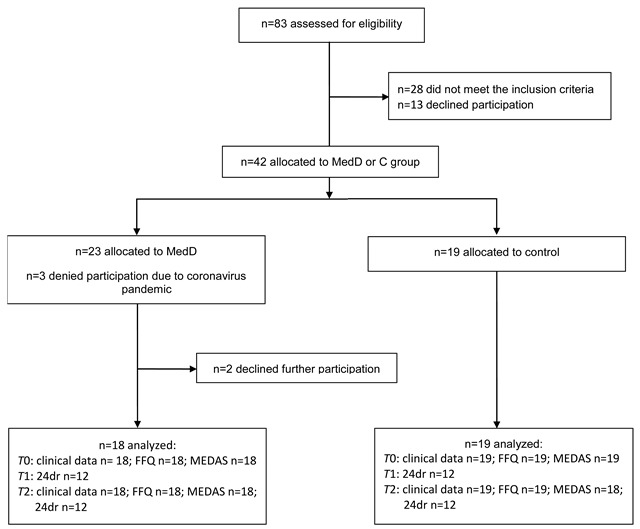


Original Figure 4:


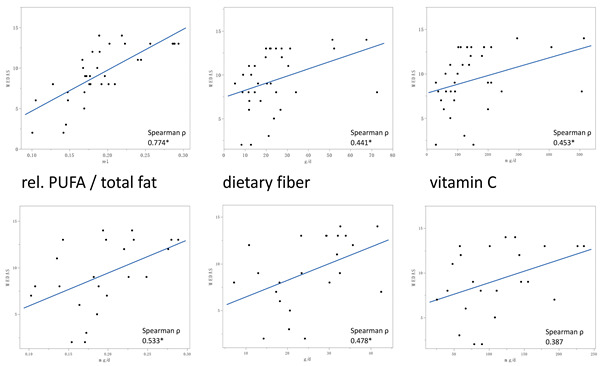


New Figure 4:


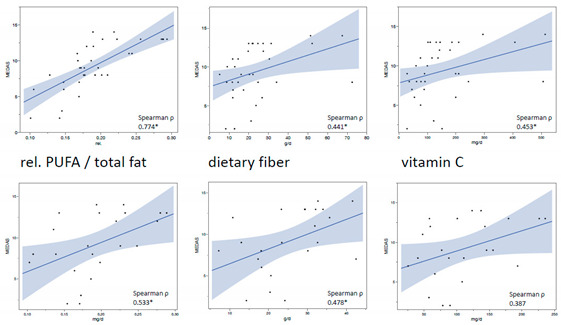


The authors apologize for any inconvenience caused and state that the scientific conclusions are unaffected. The original publication has also been updated.

## References

[1] Bartha V., Exner L., Meyer A.-L., Basrai M., Schweikert D., Adolph M., Bruckner T., Meller C., Woelber J.P., Wolff D. (2022). How to Measure Adherence to a Mediterranean Diet in Dental Studies: Is a Short Adherence Screener Enough? A Comparative Analysis. Nutrients.

